# Menstrual bleeding-specific quality of life in women on antiplatelet therapy

**DOI:** 10.1016/j.rpth.2025.102910

**Published:** 2025-06-02

**Authors:** Eva K. Kempers, Johanna A. van der Zande, Paula M. Janssen, Jérôme M.J. Cornette, Jolien W. Roos–Hesselink, Marieke J.H.A. Kruip

**Affiliations:** 1 Department of Hematology, Erasmus University Medical Center Rotterdam, Rotterdam, the Netherlands; 2 Department of Cardiology, Erasmus University Medical Center, Rotterdam, the Netherlands; 3 Department of Obstetrics and Gynecology, Erasmus University Medical Center, Rotterdam, the Netherlands; 4 Department of Neurology, Erasmus University Medical Center, Rotterdam, the Netherlands

**Keywords:** aspirin, clopidogrel, cross-sectional studies, menorrhagia, platelet aggregation inhibitors, quality of life

## Abstract

**Background:**

Antiplatelet therapy may affect menstrual blood loss in women of reproductive age.

**Objectives:**

To determine menstrual bleeding and related quality of life (QoL) in women on antiplatelet therapy.

**Methods:**

We performed a cross-sectional study at a tertiary care center in the Netherlands, including women on antiplatelet therapy with an active menstrual cycle and a control group of reproductive-aged women who did not use antiplatelets. Participants completed an online questionnaire containing 2 validated instruments: (1) the Menstrual Bleeding Questionnaire (MBQ) to measure menstrual bleeding-specific QoL, and (2) the Pictorial Blood Loss Assessment Chart (PBAC). Scaled MBQ scores range from 0 to 100, with higher scores indicating worse QoL. A PBAC score of 100 is generally considered indicative of heavy menstrual bleeding.

**Results:**

In total, 38 women prescribed antiplatelet drugs (median age, 44 years [IQR, 40-48]) and 100 control women (median age, 35 years [IQR, 27-43]) completed the study questionnaire. Most common indication for antiplatelet therapy was stroke/transient ischemic attack (26%). Acetylsalicylic acid/carbasalate calcium (50%) and clopidogrel (37%) were most frequently used. Mean (SD) scaled MBQ scores were 18.9 (11.2) among antiplatelet drug users and 22.4 (10.9) in control women (adjusted mean difference, −3.28 [95% CI, −7.90, 1.35]). Median PBAC scores were 101.5 (IQR, 50.5-207) and 96.0 (IQR, 73.0-174.8; adjusted mean ratio, 0.784 [95% CI, 0.521, 1.18]), respectively. A PBAC score >100 was reported in 37% and 36% of the antiplatelet and control groups, respectively.

**Conclusion:**

Menstrual bleeding-specific QoL was comparable between women on antiplatelet therapy and controls, although controls experienced a high burden of menstrual bleeding-related complaints. Menstrual blood loss did not seem to be increased.

## Essentials

• Antiplatelet therapy in women of reproductive age might affect menstrual blood loss.
• We determined menstrual blood loss and quality of life in women with and without antiplatelet use.
• Menstrual bleeding-specific quality of life was comparable between the 2 groups of women.
• Menstrual blood loss was also comparable and not increased compared with reference values.

## 1 Introduction

Young women, aged <45 years, are disproportionately affected by cardiovascular diseases, such as stroke, transient ischemic attack, and myocardial infarction, compared with young men [[1], [2], [3]]. After such an event, patients are often treated with aspirin or nonaspirin antiplatelet therapy, such as P2Y12 inhibitors, to reduce the recurrence risk [2,4].

Antiplatelet drugs increase overall bleeding risks, with major and minor bleeding rates comparable with those reported for anticoagulant therapy [5,6]. We previously reported that effects of antiplatelet drugs on menstrual blood loss are not well characterized, although some studies suggested that antiplatelet therapy may increase menstrual blood loss in a minority of women [7]. Multiple studies have demonstrated that anticoagulant drugs (direct oral anticoagulants [DOACs] or vitamin K antagonists [VKAs]) are associated with an increased risk of heavy menstrual bleeding (HMB) and associated negative impact on menstrual bleeding-specific quality of life (QoL) [8,9]. The current definition of HMB, proposed by the National Institute for Health and Clinical Excellence, is “*excessive menstrual blood loss that interferes with a woman’s physical, emotional, social, and/or material quality of life*” [10,11]. Menstrual blood loss assessment tools should, therefore, not only quantify menstrual blood loss but also capture QoL related to menstrual blood loss and patient experiences [12]. Therefore, we performed a cross-sectional study to determine the menstrual bleeding-specific QoL of women receiving antiplatelet therapy in comparison with a control group of reproductive-aged women.

## 2 Methods

### 2.1 Study population

We conducted a cross-sectional study between January 2024 and January 2025 among women on antiplatelet therapy, who were outpatients at the Erasmus University Medical Center, Rotterdam. This is an academic tertiary care center in the Netherlands. Women aged between 16 and 50 years who had a registered prescription for any type of antiplatelet drug in their electronic health record for any indication, without a concomitant anticoagulant prescription, were eligible for participation. Anatomical Therapeutic Chemical codes used to identify antiplatelet and anticoagulant prescriptions are provided in Supplementary Table S1. Additional inclusion criteria were examined through the online study questionnaire and included an active menstrual cycle, with or without contraception of any type, and ability to understand Dutch. Exclusion criteria were use of anticoagulants, such as DOACs or VKAs, change of contraception or intrauterine device (IUD) use ≤6 months before recruitment (ie, initiation or discontinuation), known inherited bleeding disorder, or postpartum status (up to 3 months).

Potentially eligible women were approached on behalf of their treating physician by email or telephone. We asked them whether they were interested in participating in an online questionnaire. Recruitment was restricted to the departments of cardiology, neurology, and internal medicine. Digital informed consent was obtained through the online questionnaire and was required to proceed with study participation.

Similarly, a control group of women was recruited via both social media platforms of the Erasmus University Medical Center and participants (ie, female nonrelatives), and the same eligibility criteria were applied, except for antiplatelet drug use. Details of the sample size calculation are provided in the Supplementary Methods.

### 2.2 Data collection

Data were collected via Castor EDC [13]. The study questionnaire contained 2 validated instruments to collect information on menstrual blood loss and menstrual bleeding-specific QoL: the Pictorial Blood Loss Assessment Chart (PBAC) [14,15] and the Menstrual Bleeding Questionnaire (MBQ) [16], respectively. Menstrual blood loss during one cycle was prospectively measured in a semiquantitative manner by means of the PBAC score, which is computed by summing and multiplying the number of sanitary pads and/or tampons by a factor depending on the degree to which the items were stained [15]. All participants received an instruction sheet for filling in the PBAC. A PBAC score of 100 is considered indicative of 80 mL of menstrual blood loss and is often used as a cutoff to define HMB, although optimal cutoff values vary [14,17]. The MBQ is a patient-reported outcome instrument for HMB and consists of 20 questions concerning the perception of bleeding heaviness, irregularity, pain, and impact on social and daily life [12,16]. The resulting MBQ score ranges from 0 to 75, with higher scores indicating a worse QoL [16]. MBQ scores were multiplied by 1.32 to scale them from 0 to 100 [12], enhancing comparison with studies exclusively reporting scaled MBQ scores.

Demographics, indications for antiplatelet therapy, use of oral contraceptives, IUD, history of iron deficiency and anemia, comorbid conditions, and comedications were also collected via the online questionnaire. For patients who provided additional informed consent, information on antiplatelet drug type, comorbidities, and concomitant medication prescriptions was also extracted from their electronic health records to complement the survey data. Finally, antiplatelet drug users were shortly asked about their experiences and wishes regarding care for menstrual-related complaints.

Reminders were sent after 2 weeks and 2 months in case of non- or incomplete response. In addition, antiplatelet drug users who had not yet completed the questionnaire were approached by telephone.

### 2.3 Ethical approval

This study received ethical approval from the Medical Ethical Committee of the Erasmus University Medical Center (MEC-2023-0363), and all participants provided informed consent.

### 2.4 Data analysis

Categorical variables were described as numbers and percentages. Continuous data were presented as means with SD or median with IQR (first-third quartiles). Normality was assessed with histograms and quantile-quantile plots. Participants who used a menstrual cup for ≥1 day(s) were excluded from the PBAC score calculations. In these cases, it was not possible to compute a total PBAC score since the current PBAC is neither validated nor intended to be used for menstrual cups [18]. Baseline characteristics and outcomes were compared between groups with the Wilcoxon rank sum test for continuous variables and Fisher’s exact test for categorical variables. Additionally, differences in MBQ and PBAC scores between groups were expressed by mean difference or mean ratio with corresponding 95% CIs, obtained by linear regression analyses. The crude model only included antiplatelet drug use with no use as a reference; model 2 was additionally adjusted for age and combined hormonal contraceptive (CHC) or hormonal IUD use. Scores that showed a skewed distribution were transformed by natural logarithm to obtain a closer-to-normal distribution, and coefficients with corresponding 95% CIs were exponentiated for interpretation on the original scale. Analyses were performed in R version 4.4.1 [19], with the packages *dplyr* [20], *stringr* [21], *lubridate* [22], *ggplot2* [23]*,* and *gridExtra* [24].

## 3 Results and Discussion

### 3.1 Study participants

In total, 456 women with a registered antiplatelet drug prescription, without concomitant anticoagulant prescription, were identified and approached by email. Most of these patients were pregnant and used antiplatelet therapy for prevention of (pre)eclampsia and were, therefore, ineligible for study participation. Sixty women expressed their interest and subsequently received the study questionnaire. After applying the eligibility criteria, 9 patients were excluded, 11 patients did not respond, and 2 patients were classified in the control group as they reported no antiplatelet drug use. In total, 38 women on antiplatelet therapy were included (78% of eligible participants).

Similarly, 138 women expressed interest after a call on social media or through recruitment via study participants, of whom 15 were excluded, and 25 did not fill in the survey. The final control group consisted of 100 participants (80% of eligible participants).

Antiplatelet drug users had a median age of 44 years (IQR, 40-48), and controls had a median age of 35 years (IQR, 27-43; Table 1). Most patients used acetylsalicylic acid/carbasalate calcium (50%), followed by clopidogrel (37%); these drugs were already used for a median of 2.6 years (IQR, 0.8-6.6). The most commonly reported indications for antiplatelet therapy were stroke/transient ischemic attack (26%) or a combination of indications (18%). In total, 42% of patients on antiplatelet therapy reported a history of iron deficiency and/or anemia, and 29% used any kind of hormonal contraceptives. These were 19% and 23% among the controls, respectively. The majority of participants used analgesics during menstruation (58% of antiplatelet drug users and 71% of controls), primarily paracetamol.

**Table 1:** Baseline characteristics.

Variable	Antiplatelet drug users (*n* = 38), *n* (%)^a^	Controls (*n* = 100), *n* (%)^a^	*P* value^b^
**Age (y)**			<.001
Median (IQR)	44.0 (40.0, 48.0)	35.0 (27.0, 43.0)	
*Missing*	1 (2.6)	0	
**BMI**			.60
Mean (SD)	25.2 (5.81)	25.4 (5.28)	
*Missing*	1 (2.6)	0	
**Parity**			.44
≥1	18 (47)	40 (40)	
Median (IQR)	2 (1, 2)	2 (2, 3)	
*Missing*	1 (2.6)	1 (1.0)	
**Antiplatelet drug type**^c^			
Acetylsalicylic acid/carbasalate calcium	19 (50)	−	
Clopidogrel	14 (37)	−	
DAPT	5 (13)	-	
**Indication of antiplatelet therapy**			
Stroke/TIA	10 (26)	−	
Coronary artery disease	2 (5.3)	−	
Congenital heart disease	1 (2.6)	−	
Myocardial infarction	2 (5.3)	−	
Heart transplantation	2 (5.3)	−	
Peripheral arterial disease	1 (2.6)	−	
SLE	2 (5.3)	−	
Essential thrombocythemia	2 (5.3)	−	
Multiple	7 (18)	−	
Other	8 (21)	−	
*Missing*	1 (2.6)	−	
**Duration of antiplatelet drug use (y)**			
Median (IQR)	2.6 (0.8, 6.6)	−	
*Missing*	3 (7.9)	−	
**Contraceptive use**			
CHC	5 (13)	20 (20)	.46
Progesterone-only pill	2 (5.3)	0	.07
Hormonal IUD	4 (11)	3 (3.0)	.10
Copper IUD	0	6 (6.0)	.18
*Missing*	1 (2.6)	1 (1.0)	
**Menarche age (y)**			.07
Mean (SD)	13.9 (1.57)	13.0 (1.51)	
*Missing*	3 (7.9)	1 (1.0)	
**Estimation of the average duration of menstruation (d)**			.69
Mean (SD)	6.0 (3.1)	5.5 (2.1)	
*Missing*	3 (7.9)	1 (1.0)	
**Intermenstrual bleeding**			.70
*n* (%)	9 (24)	22 (22)	
*Missing*	3 (7.9)	1 (1.0)	
**Drug use during menstruation**			<.001
NSAID	2 (5.3)	17 (17)	
Paracetamol	16 (42)	19 (19)	
Tranexamic acid	1 (2.6)	0	
Multiple	4 (11)	35 (35)	
No	12 (32)	27 (27)	
*Missing*	3 (7.9)	2 (2.0)	
**Comorbidity**			
History of iron deficiency	12 (32)	19 (19)	.11
History of anemia	12 (32)	11 (11)	.008
Endometriosis	2 (5.3)	5 (5.0)	>.99
Myoma	4 (11)	3 (3.0)	.09
PCOS	1 (2.6)	5 (5.0)	>.99
History of myocardial infarction	6 (16)	0	<.001
History of stroke/TIA	16 (42)	0	<.001
History of VTE	3 (7.9)	2 (2.0)	.13
Peripheral arterial disease	2 (5.3)	0	.07
Other	9 (24)	3 (3.0)	<.001
**Concomitant medication**			
Iron	6 (16)	0	<.001
SSRI	7 (18)	2 (2.0)	.002
Lipid lowering	16 (42)	2 (2.0)	<.001
ACE inhibitor	10 (26)	0	<.001
Calcium antagonist	7 (18)	0	<.001
Beta-blocker	7 (18)	1 (1.0)	<.001
Diuretics	5 (13)	0	.001
Steroids	7 (18)	9 (9.0)	.14
Thyroid hormone	3 (7.9)	2 (2.0)	.13
Immunosuppressives/anti-inflammatory	12 (32)	0	<.001

ACE, angiotensin-converting enzyme, BMI, body mass index; CHC, combined hormonal contraceptives; DAPT, dual antiplatelet therapy; IUD, intrauterine device; NSAID, nonsteroidal anti-inflammatory drug; PCOS, polycystic ovary syndrome; SLE, systemic lupus erythematosus; SSRI, selective serotonin reuptake inhibitor; TIA, transient ischemic attack; VTE, venous thromboembolism.

a Unless otherwise specified.

b Wilcoxon rank sum test; Fisher’s exact test.

c Doses used were acetylsalicylic acid 80 mg, carbasalate calcium 100 mg, and clopidogrel 75 mg.

### 3.2 Menstrual bleeding-specific QoL

**Figure 1:**
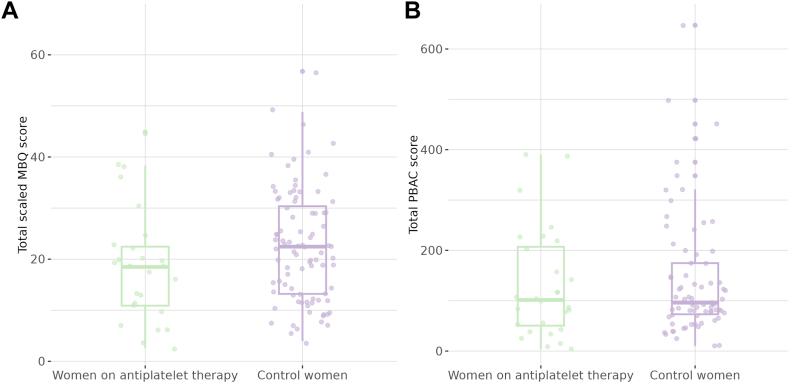
Menstrual Bleeding Questionnaire (MBQ) and Pictorial Blood Loss Assessment Chart (PBAC) scores in women on antiplatelet therapy and controls. (A) The total (unscaled) MBQ score ranges from 0 to 75, with higher scores indicating worse quality of life. Scores are multiplied by 1.32 to scale them from 0 to 100. (B) The PBAC score is computed by summing and multiplying the number of sanitary pads and/or tampons by a staining factor during one menstrual cycle. IQRs are indicated by the boxes, with the inner line displaying the median; whiskers extend up to 1.5∗ IQR. Individual data points are plotted.

The mean (SD) scaled MBQ scores were 18.9 (11.2) among antiplatelet drug users and 22.4 (10.9) among controls (adjusted mean difference, −3.28 [95% CI, −7.90, 1.35]; Figure 1A, Table 2). When additionally stratified by CHC or hormonal IUD use, mean scores were higher in both groups among women reporting no contraceptive use (Supplementary Table S2).

**Table 2:** Menstrual bleeding-specific quality of life.

Variable	Antiplatelet drug users (*n* = 38)	Controls (*n* = 100)
**Total scaled score**	*P* = .12^a^	
Mean (SD)	18.9 (11.2)	22.4 (10.9)
Median (IQR)	18.5 (10.9, 22.4)	22.4 (13.2, 30.4)
*Missing, n* (%)	12 (32)	11 (11)
Mean difference (95% CI)^b^, crude model	−3.50 (−8.35 to 1.36)	
Mean difference (95% CI), model 2^c^	−3.28 (−7.90 to 1.35)	
**Total unscaled score**		
Median (IQR)	14.0 (8.3, 17.0)	17.0 (10.0, 23.0)
Mean (SD)	14.3 (8.5)	17.0 (8.3)
*Missing, n* (%)	12 (32)	11 (11)
**Score for specific domains**		
Bleeding heaviness, median (IQR)	5.0 (3.3, 7.0)	6.0 (3.0, 8.0)
Impact on daily and social life, median (IQR)	4.0 (1.0, 8.0)	7.0 (4.0, 11.0)
**Estimates from the literature**		
Classified based on experienced MBL	Matteson et al. [16], mean unscaled MBQ score: • Normal (*n* = 88): 10.6 (SD, 8.6; IQR, 4.0-15.5) • HMB (*n* = 36): 30.8 (SD, 14.0; IQR, 23.0-37.5) Rezende et al. [25], mean unscaled MBQ score: • No AUB (*n* = 100): 7.2 ± 5.7 SD • AUB (*n* = 100): 40.1 ± 7.3 SD Rodpetch et al. [26], mean unscaled MBQ score: • Normal (*n* = 83): 15.4 ± 5.6 SD • HMB (*n* = 35): 30.4 ± 9.4 SD	
In women on anticoagulant therapy	Patel et al. [8], median scaled MBQ score: • DOAC/VKA, first cycle: 26 (IQR, 16, 41) • DOAC/VKA, second cycle: 25 (IQR, 15, 35) Hassan et al. [27], median unscaled MBQ score: • Warfarin (*n* = 30): 10 (IQR, 13) • Rivaroxaban (*n* = 27): 29.5 (IQR, 13.5)	

The total (unscaled) MBQ score ranges from 0 to 75, with higher scores indicating worse quality of life. Scores are multiplied by 1.32 to scale from 0 to 100.

AUB, abnormal uterine bleeding; DOAC, direct oral anticoagulant; HMB, heavy menstrual bleeding; MBL, menstrual blood loss; MBQ, Menstrual Bleeding Questionnaire; VKA, vitamin K antagonist.

a Wilcoxon rank sum test.

b Mean difference was calculated with no antiplatelet therapy as reference.

c Model 2 adjusted for age and use of combined hormonal contraceptives or a hormonal intrauterine device.

In comparison with the literature, mean (SD) unscaled MBQ scores reported in the initial validation study were 10.6 (8.6) among women reporting no problems with menstrual bleeding and 30.8 (14.0) among those reporting heavy menses [16]. MBQ scores have also been reported for women receiving anticoagulants. The median scaled MBQ scores among women who were prescribed a DOAC or VKA were 26 (IQR, 16-41) and 25 (IQR, 15-35) for the first and second consecutive menstrual cycle during the study period, respectively [8]. Furthermore, a South African study reported a median unscaled MBQ score of 10 (IQR, 13) among female warfarin users and 29.5 (IQR, 13.5) among rivaroxaban users [27]. Studies reporting MBQ scores for women experiencing either heavy or normal menstrual bleeding are described in Table 2. Hence, the MBQ scores in our cohort of women on antiplatelet therapy were lower than those reported for women prescribed anticoagulants and those reporting HMB, but seemed to be higher than among women without complaints. In addition, MBQ scores of the controls also seemed to be higher than those among women without complaints, and suggest that the control group concerned a selected group of women who might experience a high burden of menstrual bleeding-related complaints.

### 3.3 Pictorial blood loss assessment chart

The total median PBAC score among antiplatelet drug users was 101.5 (IQR, 50.5-207), and 37% of patients had a score > 100 (Figure 1B, Table 3). These estimates were comparable to those of control women: 96.0 (IQR, 73.0-174.8; adjusted mean ratio, 0.784 [95% CI, 0.521, 1.18]) and 36%, respectively. Hence, the mean PBAC score in the antiplatelet group was 0.8 times the mean score in the control group after adjustment for age and hormonal CHC/IUD use. Median PBAC scores were higher in women reporting no contraceptive use in both the antiplatelet (111.5 vs 36.0) and control groups (113.5 vs 77.0; Supplementary Table S3).

**Table 3:** Pictorial Blood Loss Assessment Chart scores.

Variable	Antiplatelet drug users (*n* = 38)	Controls (*n* = 100)
**Total PBAC score**	*P* = .6^a^	
Median (IQR)	101.5 (50.5, 207)	96.0 (73.0, 174.8)
*Missing, n* (%)	10 (26)	26 (26)
Mean ratio (95% CI)^b^, crude model	0.796 (0.538-1.18)	
Mean ratio (95% CI), model 2^c^	0.784 (0.521-1.18)	
**PBAC score > 100**		
*n* (%)	14 (37)	36 (36)
**Estimates from the literature**		
Classified based on experienced MBL	Reid et al. [28], women with subjective HMB (*n* = 103): • Median PBAC 222 (range, 61-545) • Median MBL measured by alkaline hematin method 95.5 mL (range, 10.2-389.4) Hald and Lieng [15], median PBAC score: • Subjective minimal MBL (*n* = 49): 45.0 (range, 2-204) • Subjective normal MBL (*n* = 168): 116.0 (range, 13-610) • Subjective HMB (*n* = 208): 254.5 (range, 13-1740) Zakherah et al. [29]: • Subjective normal MBL (*n* = 30): PBAC 120 (range, 16-460); MBL measured by alkaline hematin method 48.4 mL (range, 0.4-192.5) • Subjective HMB (*n* = 170): PBAC 225 (range, 16-674); MBL measured by alkaline hematin method 147.3 mL (range, 11.6-420.8)	
In women on anticoagulant therapy	De Jong et al. [9]: • DOAC/VKA (*n* = 98): median PBAC score during first cycle after VTE diagnosis 95 (IQR, 27-248) Patel et al. [8], median (min-max) PBAC score: • Rivaroxaban (*n* = 24): first cycle 186 (13-1232); second cycle 137 (14-1038) • Apixaban (*n* = 17): first cycle 146 (2-972); second cycle 77 (6-1038) • Warfarin (*n* = 8): first cycle 96 (17-155); second cycle 121 (51-188) Grandone et al. [30], median PBAC score: • Prior to anticoagulant therapy (*n* = 110): 120 (IQR, 65-287) • After ≥3 mo DOAC/VKA (*n* = 110): 301 (IQR, 94-443)	

The PBAC score is computed by summing and multiplying the number of sanitary pads and/or tampons by a staining factor during one menstrual cycle.

DOAC, direct oral anticoagulant; HMB, heavy menstrual bleeding; MBL, menstrual blood loss; min-max, minimum-maximum; PBAC, Pictorial Blood Loss Assessment Chart; VKA, vitamin K antagonist; VTE, venous thromboembolism.

a Wilcoxon rank sum test.

b Mean ratio derived from a linear regression model with the natural logarithm of the total PBAC score as response and no antiplatelet therapy as reference. Coefficients and corresponding 95% CIs were exponentiated for interpretation on the original scale.

c Model 2 adjusted for age and the use of combined hormonal contraceptives or a hormonal intrauterine device.

Multiple studies have been performed to validate the PBAC and determine optimal cutoff values (Table 3). One of these validation studies reported PBAC scores for 429 women who subjectively classified their menstrual blood loss as minimal, normal, or heavy [15]. Median PBAC scores for these 3 categories were 45.0 (range, 2-204), 116.0 (range, 13-610), and 254.5 (range, 13-1740), respectively [15]. Similarly, an Egyptian study reported a median PBAC score of 225 (range, 16-674) with corresponding median menstrual blood loss, measured by the alkaline hematin method, of 147.3 mL (range, 11.6-420.8) among women reporting heavy bleeding [29]. In comparison, median score was 120 (range, 16-460), and corresponding estimated blood loss was 48.4 mL (range, 0.4-192.5) among women who experienced normal menstrual bleeding [29].

PBAC scores have also been assessed in women on oral anticoagulants. Among women in whom anticoagulant therapy was initiated, the median PBAC score during the first menstrual cycle after venous thromboembolism diagnosis was 95 (IQR, 27-248) [9]. Over time, median PBAC scores decreased, ranging from 54 to 74 [9]. Furthermore, a retrospective cohort study among 110 women who were prescribed oral anticoagulants (44% DOAC, 56% VKA) for first or recurrent venous thromboembolism reported PBAC scores both for the last menstrual cycle before diagnosis and after a minimum of 3 months of anticoagulant treatment [30]. Prior to anticoagulant treatment, the median PBAC score was 120 (IQR, 65-287) and increased during treatment to 301 (IQR, 94-443) [30].

In comparison with these estimates from the literature and our control group, PBAC scores in women receiving antiplatelets do not seem to be increased and seem to be at least comparable with scores among women who self-report normal menstrual bleeding. Nevertheless, variation in PBAC scores between studies might also partly be attributed to use of different brands and sizes of sanitary pads and tampons [15]. Because most studies did not use standardized products, comparing PBAC scores between studies is not straightforward. Other disadvantages of the PBAC score include the large interindividual variation [15,28] and lack of validation of modern sanitary products with increased absorption, menstrual cups, and period underwear [18].

### 3.4 Experiences related to care of menstrual complaints

Ten women who used antiplatelet drugs (26%) indicated having spoken to a healthcare professional about menstrual blood loss, often their general practitioner or medical specialist. Four patients reported that the use of contraceptives was discussed as possible approach to decrease menstrual bleeding-related complaints, of whom 3 reported that their contraceptive use was adjusted accordingly. One woman reported shorter antiplatelet treatment duration because of menstrual bleeding-related complaints. The remaining 24 patients (63%) indicated that they did not discuss menstrual bleeding, of whom 10 patients indicated that they would like to discuss these complaints with a healthcare professional.

We previously described that the effects of antiplatelet drugs on menstrual blood loss and related QoL are currently not well characterized [7]. Estimates from the literature suggest that in a minority of women, menstrual blood loss volume, duration or perceived intensity may increase during antiplatelet therapy, but in general, conflicting results have been published [7]. Our study should be considered as a first effort to address these knowledge gaps. Nevertheless, several limitations of our study should be taken into account. As we primarily included prevalent antiplatelet drug users, we may have selected women who tolerate the drug well [31]. Also, in patients who experienced complaints related to menstrual blood loss, therapies to mitigate these consequences may have been initiated, such as the use of CHC and nonsteroidal anti-inflammatory drugs for pain relief. Furthermore, we cannot rule out that selection bias may have occurred in our study since perceived intensity of menstrual blood loss may have affected the decision to participate, both for antiplatelet drug users and controls. This is also supported by the high proportion of women reporting analgesic use during menstruation and the relatively high MBQ score for the domain of impact on daily and social life in the control group (Table 2). In addition, the MBQ score was missing for 32% of women on antiplatelet therapy compared with 11% of controls. Other study limitations include the small sample size and the lack of an intraindividual comparison. Ideally, menstrual blood loss and related QoL should be assessed among female patients before, during, and after receiving antiplatelet therapy or among patients who switch from dual to single antiplatelet therapy or *vice versa*.

In conclusion, menstrual bleeding-specific QoL was comparable between women on antiplatelet therapy and controls, although the control group consisted of a selected group of women who experienced a high burden of menstrual bleeding-related complaints. Menstrual blood loss, measured semiquantitatively by the PBAC, did not seem to be increased in women on antiplatelet therapy. Awareness of menstrual bleeding-related complaints and their impact on QoL should be increased in daily clinical practice. As we only studied a small number of prevalent antiplatelet drug users, future studies should be performed to prospectively assess menstrual blood loss and related QoL in this patient group, particularly among females initiating antiplatelet therapy. In addition, possible effects of menstrual bleeding-related complaints on therapy adherence should be examined.
