# Supplementary material for: Menstrual bleeding-specific quality of life in women on antiplatelet therapy
Source: Res Pract Thromb Haemost. 2025 Jun 2;9(4):102910. doi: 10.1016/j.rpth.2025.102910 (PMC12240165; doi:10.1016/j.rpth.2025.102910)
Supplement: Supplementary Tables [file mmc1.pdf]

Contents

Supplementary methods 2
Supplementary Table S1. ATC codes to identify antiplatelet and anticoagulant prescriptions 2
Sample size calculation 2
Supplementary results 3
Supplementary Table S2. Menstrual bleeding-specific quality of life stratified by antiplatelet therapy and contraception use 3
Supplementary Table S3. Pictorial blood loss assessment scores stratified by antiplatelet therapy and contraception use 3
References 4

Supplementary methods

Supplementary Table S1. ATC codes to identify antiplatelet and anticoagulant prescriptions

Variables	Code version	Code(s)
**Antithrombotic therapy prescriptions**		
Antiplatelet drugs	ATC	B01AC04, B01AC06, B01AC07, B01AC08, B01AC22, B01AC24, B01AC30, B01AC56
Anticoagulant drugs	ATC	B01AA, B01AB, B01AE, B01AF, B01AX05, B01AX06

Sample size calculation

Sample size calculation was based on menstrual bleeding questionnaire (MBQ)-scores reported by Rodpetch et al[1]. This study reported mean unscaled scores (SD) of 30.4 (9.4) among patients subjectively reporting heavy menstrual bleeding (HMB) (N=35) compared to 15.4 (5.6) among those who reported normal menstrual bleeding (N=83)[1]. In addition, among women (N=49) receiving oral antithrombotics, including oral anticoagulants and antiplatelet drugs, the mean (SD) in patients with HMB versus no HMB was 29.5 (2.3) and 13.9 (0.7), where HMB was defined by a MBQ-score ≥21.5[1].

We assumed a mean MBQ-score (SD) in the control group of 15 (10) and 25 (15) in the antiplatelet group with a 2:1 ratio of control to antiplatelet. For the detection of a mean difference of 10 in the MBQ-score between women on antiplatelet therapy and women who do not use these drugs, with a power of 80% and alpha of 0.05, we had to include at least 80 women in total of whom at least 27 used antiplatelet drugs.

Supplementary results

Supplementary Table S2. Menstrual bleeding-specific quality of life stratified by antiplatelet therapy and contraception use

Total scaled MBQ score	**Antiplatelet drug users** (N=38)	**Control** (N=100)
**CHC/hormonal IUD**	N=9	N=23
Median (IQR)	11.9 (4.6, 21.1)	13.2 (8.6, 22.4)
Mean (SD)	12.5 (9.2)	15.8 (10.3)
Missing, N (%)	3 (33)	0 (0)
**No CHC/hormonal IUD**	N=28	N=76
Median (IQR)	19.1 (12.9, 26.4)	23.8 (17.2, 31.7)
Mean (SD)	20.8 (11.2)	24.2 (9.5)
Missing, N (%)	8 (29)	11 (14)

The total (unscaled) menstrual bleeding questionnaire (MBQ) score ranges from 0-75, with higher scores indicating worse quality of life. Scores are multiplied by 1.32 to scale from 0-100.
Abbreviations:
CHC = combined hormonal contraceptives; IQR = interquartile range; IUD = intra-uterine device; MBQ = menstrual bleeding questionnaire; SD = standard deviation.

Supplementary Table S3. Pictorial blood loss assessment scores stratified by antiplatelet therapy and contraception use

Total PBAC score	**Antiplatelet drug users** (N=38)	**Control** (N=100)
**CHC/hormonal IUD**	N=9	N=23
Median (IQR)	36.0 (27.3, 70.3)	77.0 (62.5, 93.8)
Missing, N (%)	3 (33)	7 (30)
**No CHC/hormonal IUD**	N=28	N=76
Median (IQR)	111.5 (79.5, 221)	113.5 (80.5, 196.5)
Missing, N (%)	6 (21)	18 (24)

Pictorial blood loss assessment chart (PBAC) score is computed by summing and multiplying the number of sanitary pads and/or tampons by a staining factor during one menstrual cycle.
Abbreviations:
CHC = combined hormonal contraceptives; IQR = interquartile range; IUD = intra-uterine device; PBAC = pictorial blood loss assessment chart; SD = standard deviation.

References

1. Rodpetch T, Manonai J, Angchaisuksiri P, Boonyawat K. A quality-of-life questionnaire for heavy menstrual bleeding in Thai women receiving oral antithrombotics: Assessment of the translated Menstrual Bleeding Questionnaire. *Res Pract Thromb Haemost* 2021;**5**:e12617. doi: 10.1002/rth2.12617.
